# Multidimensional Profiles of Religiosity Among Grandparents, Parents, and Grandchildren

**DOI:** 10.1093/geroni/igaa057.1649

**Published:** 2020-12-16

**Authors:** Joonsik Yoon, Woosang Hwang, Merril Silverstein, Maria Brown

**Affiliations:** Syracuse University, Syracuse, New York, United States

## Abstract

Studies have frequently treated different measures of religiosity as cumulative components on a single scale of religiosity ranging in value from high to low, despite the broad consensus that religiosity is multidimensional in nature. Multidimensional typologies can be helpful not only in properly assessing and measuring religiosity in an increasingly diverse population, but could also help identify specific religious and spiritual differences within seemingly similar groups. The different dimensions of religiosity also allow us to classify populations according to their religious characteristics rather than according to social outcomes in relation to their religiosity. We apply latent class analysis and multiple group latent class analysis to multigenerational data from the Longitudinal Study of Generations (N= 1,726) to examine (1) what religiosity profiles exist across three generations (grandparents, parents, and grandchildren), and (2) how the patterns of religiosity profiles are the same or different across three generations. Results of the latent class analysis show that four religiosity subgroups are identified in the three generations: strongly religious, weakly religious, religious but not literalists, literalists but not religious. In addition, results of the multigroup latent class analysis show that the four religiosity subgroups are structurally the same across three generation. Our findings complement previous studies that show religiosity is a multidimensional construct, and demonstrate that religiosity class memberships are invariant across three generations. Furthermore, given that one in four U.S. adults consider themselves “spiritual but not religious” or “religious but not spiritual”, our findings confirm similar patterns of religiosity subgroups in three generations.

